# Spontaneous Cervical Intramedullary and Subarachnoid Hemorrhage due to a Sulco-Commissural Artery Aneurysm

**DOI:** 10.1007/s00062-019-00772-6

**Published:** 2019-03-14

**Authors:** E. Donauer, M. Aguilar Pérez, N. Jangid, B. Tomandl, O. Ganslandt, H. Henkes

**Affiliations:** 1 Klinik für Neurochirurgie und Frührehabilitation, MediClin Krankenhaus Plau am See, Quetziner Str. 88, 19395 Plau am See, Germany; 2 Neuroradiologische Klinik, Kopf- und Neurozentrum, Klinikum Stuttgart, Kriegsbergstraße 60, 70174 Stuttgart, Germany; 3 Klinik für Radiologie und Neuroradiologie, Klinikum Christophsbad, Faurndauer Straße 6–28, 73035 Göppingen, Germany; 4 Neurochirurgische Klinik, Kopf- und Neurozentrum, Klinikum Stuttgart, Kriegsbergstraße 60, 70174 Stuttgart, Germany; 5 Medizinische Fakultät, Universität Duisburg-Essen, Hufelandstraße 55, 45147 Essen, Germany

## Introduction

Aneurysms of the arteries of the spinal cord are rare. They are known to be a potential cause of hematomyelia and subarachnoid hemorrhage. Most aneurysms are associated with and secondary to another vascular lesion with an arteriovenous shunt, e. g. spinal cord arteriovenous malformation (AVM). Within this group, aneurysms of the sulco-commissural arteries are infrequently reported. Due to their location within the spinal cord, endovascular treatment is generally favored over microsurgical extirpation. This article reports the case of an otherwise healthy woman with a spontaneous hemorrhage into the spinal cord and the subarachnoid space. Digital subtraction angiography (DSA) showed an aneurysm of a cervical sulco-commissural artery as the source of this hemorrhage as well as revealing a cervical intradural arteriovenous fistula. An unsuccessful attempt was made to catheterize the aneurysm, so it was decided to perform a microsurgical extirpation of the sulco-commissural aneurysm instead. Surgical access to the aneurysm was facilitated by the previous hemorrhage; however, it would most likely also be possible for an unruptured aneurysm of this kind. This article describes in detail how this patient’s case was managed.

## Case Report

**Fig. 1:**
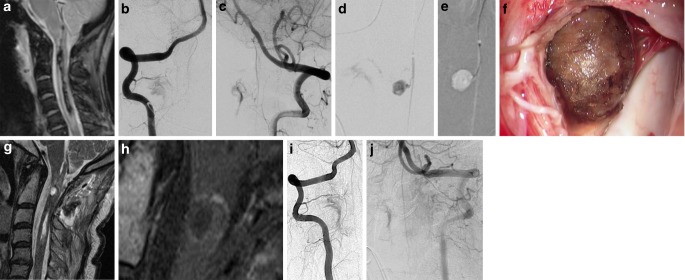
Diagnostic imaging and treatment in a patient with cervical hematomyelia due to an aneurysm of a sulco-commissural artery, associated with a cervical intradural arteriovenous fistula. T2*weighted image showing the hematoma in the cervical spinal cord and the surrounding edema (**a**). DSA at this time revealed an intradural arteriovenous fistula with dominant radiculomeningeal supply via the right VA (**b**). Injection of the left vertebral artery shows the pial supply of the intradural arteriovenous fistula via the anterior spinal artery (**c**). Selective injection of the anterior spinal artery 5 months later shows the connection between the anterior spinal artery and the aneurysm via a sulco-commissural artery (**d**). Meanwhile the aneurysm had considerably increased. While the guidewire could be introduced into the sulco-commissural artery, the vessel’s small caliber did not allow passage of the microcatheter (**e**). Via a small microsurgical approach to the cervical spinal cord from the right side and after dissection of the spinal cord tissue, the wall of the aneurysm became visible (**f**). Thermocoagulation of the wall of the aneurysm resulted in shrinkage of the lesion, providing additional space for further dissection of the whole aneurysm. After mobilization of the aneurysm, the afferent sulco-commissural artery was identified and the borders of the adjacent spinal cord were visible. The feeding artery was divided with microscissors. Postoperative follow-up MRI on the first day after the microsurgical aneurysm resection shows the hemosiderin remnants (T2-weighted image **g**) while the previous contrast enhancing aneurysm wall is no longer visible on contrast enhanced T1WI (**h**). DSA of the right (**i**) and left (**j**) vertebral artery 3 weeks after the microsurgical resection of the sulco-commissural artery aneurysm. The previous aneurysm is no longer visible, the continuity of the anterior spinal artery is preserved and a small remnant of the intradural AV-fistula remains perfused

A 56-year-old female patient presented with severe headache, tetraparesis, hemiplegia and hypoglossal paralysis on the right side and somnolence. The medical history was essentially unremarkable. Shortly after admission to the referring hospital, she became comatose. Cranial non-contrast computed tomography (NCCT) showed a hematoma in the medulla oblongata and thrombus in the fourth ventricle. Magnetic resonance imaging (MRI) revealed edema and hematoma of the medulla oblongata and the cervical spinal cord extending to the level of the 6th cervical vertebral body (Fig. 1a). The hematoma had ruptured through the floor of the fourth ventricle. At the C1 and C2 levels, a spherical structure was visible within the hematoma. A DSA was carried out on the same day and this showed an intradural arteriovenous fistula at the C2 level on the right side. Supply of this intradural fistula was from the right vertebral artery (VA) at the atlas loop (Fig. 1b) as well as from the anterior spinal artery (ASA) (Fig. 1c). A very small aneurysm was visible on a sulco-commissural artery adjacent to the ASA, located at the level of C1 and C2. The intention of the treatment was to prevent another intramedullary and/or subarachnoid hemorrhage arising from a re-rupture of the sulco-commissural artery aneurysm. During the acute posthemorrhagic phase, treatment was postponed to facilitate neurological recovery. The treatment options were discussed 5 months later when, after rehabilitation treatment, the clinical condition of the patient had considerably stabilized. The first choice was to attempt endovascular treatment. Entering via the right VA, the artery originating from the right V3 segment and supplying the intradural fistula was catheterized and injected. Embolization was not performed for two reasons: 1) there was a possibility of cervical spinal cord supply via this vessel and 2) a partial embolization with interruption of the dural supply only might have recruited pial feeding arteries and could have increased the hemodynamic stress on the aneurysm. The right V4 segment and the ASA were then carefully catheterized with three different microcatheters (Magic 1.2, Balt Extrusion, Montmorency, France; Marathon, Medtronic, Dublin, Ireland; Excelsior SL10, Stryker, Kalamazoo, MI, USA). Injecting contrast medium into the ASA illuminated the hypertrophic right-sided sulco-commissural artery and the aneurysm. From this position a part of the intradural fistula was also opacified (Fig. 1d). The use of DynaCT (Siemens, Erlangen, Germany) with contrast medium injection of the right VA confirmed the location of the aneurysm within the spinal cord. Wire access was gained along the ASA to the sulco-commissural artery in a controlled fashion. Several attempts were made to introduce a microcatheter into the aneurysm sac. Various combinations of Magic 1.2, Marathon and Excelsior SL10 with Mirage (Medtronic, Dublin, Ireland) and Hybrid (Balt Extrusion, Montmorency, France) wires were used but the small caliber of the sulco-commissural artery did not allow safe microcatheter access to the aneurysm (Fig. 1e). The failed endovascular procedure was tolerated with no new neurological deficits. The right-sided hemiplegia improved slowly over the following few weeks, which motivated the patient to undergo open surgery. The potential risks of both conservative management and surgery were explained to the patient and her family and she eventually accepted the proposed operation.

The patient underwent surgical extirpation of the aneurysm on a right-sided cervical sulco-commissural artery via a small intramedullary approach after a C0/C1 laminectomy and a partial laminectomy of C2 with a partial coagulation of the arteriovenous fistula, which turned out to be located within the dura. The dura was opened in a hinged door fashion on the right side. The dura was reflected and fixed in place, followed by further preparation within the arachnoid. A small opening was created below the cisterna magna at the C1 level caudal to the exiting dorsal nerve root. The arachnoid was opened and the denticulate ligaments were divided. The gliotic changes resulting from the previous hemorrhage 5 months ago were clearly visible in the exposed spinal cord. Hemosiderin-containing gliotic areas were present around the aneurysm and there was a persistent syringomyelia in the central spinal cord. Under 12 × magnification, a longitudinal incision of approximately 8 mm was made cephalic to the gliosis, revealing the aneurysm. Using low power, thermocoagulation was used to shrink the aneurysm and then mobilize it from the spinal cord, without damaging the adjacent nerve roots or vessels. Small tortuous vessels of the arteriovenous fistula were coagulated and obliterated. The aneurysm was further separated from the discolored parenchyma of the spinal cord and was removed after coagulating and dividing the afferent artery. The aneurysm could be removed without hemorrhage while preserving the spinal cord and the nerve roots (Fig. 1f). While resecting the aneurysm, strong muscular walls and thrombus became visible. After irrigating with saline, the dura was closed in a watertight fashion. The wound was closed in three layers. The patient was extubated immediately following the operation. The patient had a minor paresis of the left hand, which resolved within 24 h. The subsequent neurological status of the patient initially remained unchanged and the wound healed by primary intention. Inpatient rehabilitation was continued for 4 months, with slight improvement of the right side dominant tetraparesis. During a visit 6 months after surgery the clinical condition was rated mRS 4 due to a slowly improving tetraparesis which had resulted from the primary hemorrhage. The MRI performed on day 1 after the operation described above confirmed the removal of the hemosiderin remnants and of the contrast enhanced aneurysm wall within the cervical spinal cord at the C1 and 2 levels (Fig. 1g, h). The signal intensity of the previous aneurysm location on T2WI had decreased 3 weeks later while a contrast-enhancing rim of surrounding spinal cord was present. The DSA 3 weeks after surgery confirmed the obliteration of the sulco-commissural artery aneurysm and a small perfused remnant of the intradural arteriovenous fistula (Fig. 1i).

## Discussion

Aneurysms of the ASA and its branches are extremely rare and they may occur as isolated lesions without an identifiable reason [1–3]. The assumption of an underlying dissection sometimes appears to be rather speculative [3, 4]. Cervical spondylosis, per se a very frequent disorder, has been reported to be associated with the formation of an adjacent ASA aneurysm [5].

As in the case reported here, ASA aneurysms can be found in conjunction with a variety of arteriovenous shunts. They are usually not encountered in spinal arteriovenous fistulas. Biondi et al. (1992) [6, 7] as well as Konan et al. [8] described sulco-commissural aneurysms, which were located on feeding arteries of spinal cord arteriovenous malformations. In these cases both the hemodynamic stress due to the increased blood flow as well as a developmental factor may have contributed to the aneurysm formation. In ASA aneurysms associated with steno-occlusive disease of major cranial arteries, the collateral flow via the spinal vasculature is the most likely cause of aneurysm development [9–12]. More complex etiopathogenetic relationships become apparent in ASA aneurysms in the context of epidural and intradural arteriovenous fistulas. Takasaki et al. (1991) [13] clipped an ASA aneurysm in a patient with a dural arteriovenous fistula of the posterior fossa. In this case, due to the poor image quality of the available prints, it is difficult to determine if the ASA contributed to the dural arteriovenous shunt. Most likely there was no hemodynamic connection between the fistula and the aneurysm (Ryogo Anei, Department of Neurosurgery, Asahikawa Medical University, Hokkaido, Japan; personal communication). In the patient reported by Gilard et al. (2013) [14] the dural arteriovenous fistula was located at the foramen magnum and the ASA with the aneurysm supplied the fistula through the left C1 radiculopial artery. Both patients presented with an SAH. Matsui et al. (2007) [15] and Onda et al. (2012) [16] operated on patients presenting with hematomyelia due to combined dural AV shunts and spinal artery aneurysms. The AV shunt was located at the C1/C2 level and was supplied by both dural and pial feeding arteries, including the ASA. The aneurysms in both cases most likely arose from a sulco-commissural artery, because they were located lateral to the expected continuation of the ASA. Since these arteries participated in the supply of the AV shunt the aneurysms can be considered as flow-related. A patient presenting with an SAH and otherwise features almost identical to the present case was reported by Nakagawa et al. (2014) [17]. An AV fistula located at C1–C2, which was called “epidural” by the authors, was supplied from the radicular artery and from pial vessels arising from the ASA. The authors used the term “anterior spinal artery aneurysm”. The DSA images, however, show the aneurysm lateral to the expected continuation of the ASA, as would be the case in an aneurysm of the sulco-commissural artery. In the present patient, an intradural AV fistula on the right side at the C2 level was supplied by the radicular artery from the right vertebral artery as well as from several pial arteries originating from the ASA. The venous drainage was exclusively paraspinal. The assumption was that the intradural AV fistula with the radicular supply was the primary lesion. It appears at least possible that such a fistula, located *in* the dura, may recruit supply from pial vessels. A flow-related induction of the aneurysm formation seems plausible. Alternatively, a metameric or myelomeric connection between the AV shunt and the aneurysm is at least a possibility [18]. The documented growth of the aneurysms is an interesting parallel between the case of Nakagawa et al. (2014) [17] and the present case.

The management of this unusual condition is a matter of controversy and the clinical decision making must take the patient’s individual circumstances into account. A poor clinical condition or advanced age are certainly arguments in favor of conservative management, and a good outcome is possible [2, 5], even with spontaneous thrombosis of the aneurysm [3, 19]. On the other hand, re-hemorrhage and death may occur [20]. Direct surgical treatment of such aneurysms has been infrequently reported [9, 13, 15, 21, 22]. The *indirect* influence on ASA aneurysms by interrupting the AV shunt [16] or placing a flow diverter in the vertebral artery covering the origin of the ASA [23] is an interesting concept.

The aneurysm in the present patient was not located in continuity with the ASA but originated from a sulco-commissural artery, the small caliber of which made aneurysm catheterization impossible. Any further observation was discarded after the aneurysm had grown considerably over 5 months of conservative management. A transarterial embolization of the intradural arteriovenous fistula would have been technically straightforward. Obliterating an AV shunt in order to induce the shrinkage of a flow-related aneurysm is a popular concept in the treatment of pial brain AVMs. Complete proximal aneurysm obliteration is, however, more the exception than the rule [24]. The hemodynamic strategy was dismissed since an interruption of the dural supply without impact on the pial feeding arteries might have even increased the hemodynamic stress on the ASA and the unsecured aneurysm. The glial scar around the aneurysm, resulting from the previous hemorrhage, enabled the dissection of the cervical spinal cord without further damage. It was also possible to perform a resection of this aneurysm as it was separate from the ASA. The previous hemorrhage facilitated the microsurgical extirpation without further damage to the spinal cord. A microsurgical obliteration of the epidural AV fistula was attempted but was only partially successful. An embolization of the remaining dural supply of the epidural AV fistula was offered to the patient, who was hesitant to undergo further treatment for the time being.

The poor clinical outcome (mRS 4) of this patient might be an argument in favor of pro-active treatment in patients diagnosed prior to hemorrhage.
